# The antityrosinase and antioxidant activities of flavonoids dominated by the number and location of phenolic hydroxyl groups

**DOI:** 10.1186/s13020-018-0206-9

**Published:** 2018-10-19

**Authors:** Ai-Ren Zuo, Huan-Huan Dong, Yan-Ying Yu, Qing-Long Shu, Li-Xiang Zheng, Xiong-Ying Yu, Shu-Wen Cao

**Affiliations:** 1 State Key Laboratory of Food Science and Technology, Nanchang University, Nanchang, 330047 Jiangxi China; 2 Jiangxi University of Traditional Chinese Medicine, Nanchang, Jiangxi China; 3 Department of Chemistry, Nanchang University, Nanchang, Jiangxi China

**Keywords:** Antityrosinase activity, Molecular docking, Antioxidant activity, Phenolic hydroxyl, Isoeugenol, Shikonin

## Abstract

**Background:**

Compounds with the ability to scavenge reactive oxygen species (ROS) and inhibit tyrosinase may be useful for the treatment and prevention from ROS-related diseases. The number and location of phenolic hydroxyl of the flavonoids will significantly influence the inhibition of tyrosinase activity. Phenolic hydroxyl is indispensable to the antioxidant activity of flavonoids. Isoeugenol, shikonin, baicalein, rosmarinic acid, and dihydromyricetin have respectively one, two, three, four, or five phenolic hydroxyls. The different molecular structures with the similar structure to l-3,4-dihydroxyphenylalanine (l-DOPA) were expected to the different antityrosinase and antioxidant activities.

**Methods:**

This investigation tested the antityrosinase activity, the inhibition constant, and inhibition type of isoeugenol, shikonin, baicalein, rosmarinic acid, and dihydromyricetin. Molecular docking was examined by the Discovery Studio 2.5 (CDOCKER Dock, Dassault Systemes BIOVIA, USA). This experiment also examined the antioxidant effects of the five compounds on supercoiled pBR322 plasmid DNA, lipid peroxidation in rat liver mitochondria in vitro, and DPPH, ABTS, hydroxyl, or superoxide free radical scavenging activity in vitro.

**Results:**

The compounds exhibited good antityrosinase activities. Molecular docking results implied that the compounds could interact with the amino acid residues in the active site center of antityrosinase. These compounds also exhibited antioxidant effects on DPPH, ABTS, hydroxyl, or superoxide free radical scavenging activity in vitro, lipid peroxidation in rat liver mitochondria induced by Fe^2+^/vitamin C system in vitro, and supercoiled pBR322 plasmid DNA. The activity order is isoeugenol < shikonin < baicalein < rosmarinic acid < dihydromyricetin. The results showed the compounds with more phenolic hydroxyls have more antioxidant and antityrosinase activities.

**Conclusion:**

This was the first study of molecular docking for modeling the antityrosinase activity of compounds. This was also the first study of the protective effects of compounds on supercoiled pBR322 plasmid DNA, the lipid peroxidation inhibition activity in liver mitochondria. These results suggest that the compounds exhibited antityrosinase and antioxidant activities may be useful in skin pigmentation and food additives.

**Electronic supplementary material:**

The online version of this article (10.1186/s13020-018-0206-9) contains supplementary material, which is available to authorized users.

## Background

Flavonoids play a key role in the treatment of various diseases. Compounds with the ability to protect against DNA damage caused by reactive oxygen species (ROS) and inhibit tyrosinase may be useful for the treatment and prevention from ROS-related diseases. Flavonoids are a large type of compounds in natural products. Flavonoids already have been used widely as lead compounds or drugs.

Some studies showed that the number and location of phenolic hydroxyl on the flavonoids will significantly influence the inhibition of tyrosinase activity [1–3]. The number of phenolic hydroxyl on the B ring of flavonoids or catechins structure or resorcinol structure, can greatly enhance the inhibition of tyrosinase activity. At present, 4-hexyl resorcinol have been used as commodity in shrimp preservation [4]. The number and position of phenolic hydroxyl on the 1,2-diphenylethene derivatives can greatly effect the inhibition of tyrosinase activity. Two phenol hydroxyls compared to one hydroxyl and phenol hydroxyl replaced methoxyl will significantly enhance the inhibition of tyrosinase activity [5–7].

The tyrosinase inhibition mechanism of phenol hydroxyl compounds was analysed. Because the activity center of tyrosinase is hydrophobic, H ^+^, combined with Eoxy double oxygen, only come from the hydroxyl of tyrosine and dopamine. Phenol hydroxyl compounds, similar to tyrosine and dopamine, can inhibit the activity of tyrosinase [8].

Phenolic hydroxyl is indispensable to the antioxidant activity of flavonoids. Many studies showed that the antioxidant activity increased with the phenol hydroxyl number in B ring of flavonoids. Seyoum [9] studied the activity of scavenging free radicals of 52 kinds of flavonoids. The result showed that two or three phenol hydroxyls compared to one hydroxyl in A ring or B ring, will greatly enhance the antioxidant activity.

The relationship between phenolic hydroxyl number and antioxidant activity of flavonoids is very significant. The reason may be: (1) the more phenolic hydroxyl number, the more H^+^ combined with free radicals; (2) the phenolic hydroxyl has strongly denounce electronic effect, which result to the free radicals reaction; (3) the more phenolic hydroxyl number, the more hydrogen bonding, antioxidant activity is also enhanced obviously [10].

The number and location of phenolic hydroxyl of the flavonoids will significantly influence the inhibition of tyrosinase activity. Phenolic hydroxyl is indispensable to the antioxidant activity of flavonoids. Isoeugenol, shikonin, baicalein, rosmarinic acid, and dihydromyricetin have respectively one, two, three, four, or five phenolic hydroxyls. The different molecular structures with the similar structure to l-3,4-dihydroxyphenylalanine (l-DOPA) were expected to the different antityrosinase and antioxidant activities.

Tyrosinase (EC 1.14.18.1) plays a key role in the biosynthesis of melanin pigment [11]. Under normal physiological conditions, melanin plays a key role in the protection against UV injury, animal mimicry and camouflage [12]. Thus, it has attracted researchers to find efficient tyrosinase inhibitors. Recently, molecular docking for modeling the antityrosinase activity of compounds had been used widely in drug design [13].

Isoeugenol is the major constituent of *Eugenia caryophyllata* Thunb., which has extensive pharmacological activities, such as antimicrobe, stomach-invigorating. The result of Jin [14] indicated that isoeugenol analogs exhibited the cytotoxic activity against A549, KB, and KB-VCR cell lines.

Shikonin is the major constituent of *Arnebia euchroma*(*Royle*)*Johnst*, which has extensive pharmacological activities. Shikonin has good antioxidant activities, which supports the use of shikonin as the new anti-aging candidate drug, cosmetic materials and food additives. The results of Chen [15] revealed that SK-Hep-1 cells apoptosis induced by shikonin proceeds by involvement of reactive oxygen species and an oxidative stress-mediated pathway.

Baicalein, a kind of oriental medicine, exhibits antioxidant and anti-inflammatory activities. The results of Li-Weber [16] revealed that baicalein can inhibit several genes of the cell cycle, attenuate NF-κB activity, and scavenge many kinds of oxidative radicals.

Rosmarinic acid, isolated from *Perilla frutescens* (L.) or *Rosmarinus officinalis*, exhibits many potent biological activities. The result of Zhu [17] indicated that rosmarinic acid extract exhibits the high activity of inhibiting á-glucosidase for allergy treatments and diabetes mellitus.

Dihydromyricetin can be used to scavenge the free radicals. It also has the effects of anti-oxidation and anti-tumour. Based on the results of Xin [18], dihydromyricetin was less toxic and highly effective as a good, natural antioxidant for polypropylene.

This investigation tested the antityrosinase activity, the inhibition constant, and inhibition type of compounds. Molecular docking can simulate the binding mode and binding affinity of the tyrosinase and compounds. This investigation also tested the antioxidant effects of isoeugenol, shikonin, baicalein, rosmarinic acid, and dihydromyricetin on supercoiled pBR322 plasmid DNA, lipid peroxidation, and DPPH, ABTS, hydroxyl, or superoxide free radical scavenging activity in vitro.

## Methods

### Chemicals and reagents

Isoeugenol, shikonin, baicalein, rosmarinic acid, dihydromyricetin, l-3,4-dihydroxyphenylalanine (l-DOPA), tyrosinase (EC 1.14.18.1), phenanthroline, pyrogallol, 2, 2′-azino-bis (3-ethylbenzothiazoline-6-sulfonic acid) (ABTS), diphenyl-2-picrylhydrazyl (DPPH), thiobarbituric acid (TBA), and 2,2′-azobis(2-methylpropionamidine)dihydrochloride (AAPH) were purchased from the Sigma Chemical Company (St. Louis, MO, USA). C3606 reagent kit for organization mitochondria separation was purchased from Shanghai Biyuntian company. Disodium phosphate, sodium dihydrogen phosphate, K_2_S_2_O_8_, potassium sulfate, and ferrous sulfate were purchased from Sinopharm Chemical Reagent Co., Ltd (Shanghai, China). All other solvents and chemicals with analytical grade were commercially available. The Minimum Standards of Reporting Checklist contains details of the experimental design, and statistics, and resources used in this study (Additional file 1).

### Tyrosinase activity assay

According to the reference of Chen et al. [19], the tyrosinase activity was measured using l-DOPA as a substrate. Dimethyl sulfoxide (DMSO) was used to dissolve the inhibitor samples. l-DOPA in PBS buffer (pH 6.8) was previously incubated at 30 °C. Then, 0.1 mL sample was mixed with 2.8 mL l-DOPA (0.5 mM). After 1 min, the mixture was added to 0.1 mL of tyrosinase solution (5.33 μg/mL) at 475 nm for 400 s, the absorbance was immediately monitored. The relative enzyme activity was regarded as the slope of the linear part. The inhibitory concentration 50 (IC_50_) was used to examined the antityrosinase activity. Each sample was examined in five times and averaged. The inhibitory rate was examined according to the formula:

1 $${\text{Inhibitory}}\,{\text{rate}}\left( \% \right) = [({\text{S}}_{0} - {\text{S}}_{ 1} )/{\text{S}}_{0} ] \times 100\%$$

where S_1_ is the slope value with samples and S_0_ is the slope value without samples.

### Determination of the inhibition type and inhibition constant

By Lineweaver–Burk plot, the inhibition type was assayed. The inhibition constant was assayed by the second plots of the apparent K_m_/V_mapp_ or 1/V_mapp_ versus the concentration of the inhibitor.

### Molecular docking study

Molecular docking can predict the binding mode and binding affinity of the tyrosinase and compounds. From the Protein Data Bank (UCSD/SDSC and Rutgers, http://www.rcsb.org/), the crystal structure of tyrosinase (PDB code: 2Y9X) was available [20]. The polar hydrogen was added and all ligands and bound water were eliminated. The ligands were used as configuration of each compound. Using Discovery Studio Version 4.5 (CDOCKER Dock, Dassault Systemes BIOVIA, USA), molecular docking was carried out and the interactions were analyzed [21].

### DPPH free radical scavenging activity

According to the references of Lee et al. [22], DPPH free radical scavenging capacity was measured. In the tube, 1 mL of tested samples in different concentrations was added in turn. 3.5 mL of ethanol and 0.5 mL of 0.6 mmol/L DPPH methanol solution were added. In room temperature and a dark environment, the reaction lasted 30 min. The wavelength used was 517 nm. Each sample was examined in three times and averaged. The DPPH scavenging activity was examined according to the formula:

2 $${\text{DPPH}}\,{\text{scavenging}}\,{\text{activity }}\left( \% \right) = \left[ {\left( {A_{\text{C}} - A_{\text{S}} } \right)/A_{\text{C}} } \right] \times 100\%$$

where *A*_S_ is the absorbance value with samples and *A*_C_ is the absorbance value without samples.

### ABTS free radical scavenging activity

According to the references of Wan et al. [23], ABTS free radical scavenging capacity was measured. ABTS was dissolved in water to make 7 mmol/L ABTS water solution. ABTS ^+^ was produced by reacting 2.45 mmol/L potassium persulfate (K_2_S_2_O_8_) with the ABTS stock solution. The reaction lasted 12–16 h at room temperature in the dark. The absorbance of ABTS^+^ stock solution at 734 nm was 0.70 ± 0.02, diluted with methanol.

Samples (0.5 mL) were added to ABTS^+^ (5 mL) for 6 min. The control group contains 0.5 mL of ethanol and 5 mL of ABTS^+^ solution. Each sample was examined in three times and averaged. The ABTS^+^ scavenging activity was examined according to the formula:

3 $${\text{ABTS}}^{ + } \,{\text{scavenging}}\,{\text{activity }}\left( \% \right) = \left[ {\left( {A_{\text{C}} - A_{\text{S}} } \right)/A_{\text{C}} } \right] \times 100\%$$

where *A*_S_ is the absorbance value with samples and *A*_C_ is the absorbance value without samples.

### Hydroxyl free radical scavenging activity

According to the references of De Avellar IGJ et al. [24], hydroxyl free radical scavenging capacity was measured. In the tube, 0.2 mL of samples, 1 mL of PBS buffer (pH = 7.4),0.2 mL of 5 mmol/L phenanthroline, 0.2 mL of 7.5 mmol/L FeSO_4_, 0.2 mL of 0.05% H_2_O_2_, 3.2 mL of ethanol were added in turn for 20 min in 37 °C. The wavelength used was 536 nm. Each sample was examined in three times and averaged. The hydroxyl free radical scavenging activity was examined according to the formula:

4 $${\text{Hydroxyl}}\,{\text{free}}\,{\text{radical}}\,{\text{scavenging}}\,{\text{activity }}\left( \% \right) = \left[ {\left( {A_{\text{C}} - A_{\text{S}} } \right)/A_{\text{C}} } \right] \times 100\%$$

where *A*_S_ is the absorbance value with samples and *A*_C_ is the absorbance value without samples.

### Superoxide free radical scavenging activity

According to the references of Shen et al. [25], superoxide free radical scavenging capacity was measured using Varioskan Flash multifunction microplate reader (Thermo scientific, USA) and 96 wells plates. Each well was added 264 μL PBS buffer (pH = 8.2), 12 μL samples of different concentrations, 25 °C for 10 min. Then 24 μL of 1.25 mmoL/L pyrogallol solution was added and shaken 3 s quickly. The blank group is ethanol. Absorbance values were measured every 30 s. The reaction lasted 5 min in 37 °C. The wavelength used was 320 nm. Each sample was measured in triplicate and averaged. The slope is the self-oxidation rate of pyrogallol. The lower slope indicated the better superoxide free radicals scavenging capacity.

Each sample was examined in three times and averaged. The inhibitory rate was examined according to the formula:

5 $${\text{Superoxide}}\,{\text{free}}\,{\text{radical}}\,{\text{scavenging}}\,{\text{activity }}\left( \% \right) = \left[ {\left( {S_{\text{C}} - S_{\text{S}} } \right)/S_{\text{C}} } \right] \times 100\%$$

where *S*_C_ is the slope value without samples and *S*_S_ is the slope value with samples.

### Lipid peroxidation assay in liver mitochondria in vitro

Using the diagnostic kits from Biyuntian (Shanghai, China), liver mitochondria were obtained. The liver mitochondria from Sprague–Dawley (SD) rats were obtained, according to the references of Zuo et al. [26].

In the tubes, 1 mL of mitochondria liquid, 0.5 mL of antioxidant solution, 0.25 mL of 1 mM Vitamin C, and 0.25 mL of 0.1 mM Fe^2+^ were added in turn. The positive control group contains 0.5 mL of 0.05 M PBS buffer, instead of the antioxidant solution. The blank group was added 1 mL of mitochondrial liquid and 1 mL of 0.05 M PBS buffer. The reaction lasted for 1 h at 37 °C. 2.5% hydrochloric acid solution and 2 mL of 20% CCl_3_COOH were added for 10 min, followed by 0.3% NaOH solution and 2 mL of 0.67% TBA were added. The test tubes were placed in the water for 30 min at 95 °C, then centrifuged for 10 min at 1372*g*. The wavelength used was 532 nm. Each sample was examined in three times and averaged. The lipid peroxidation inhibition activity was examined according to the formula:

6 $${\text{Lipid\,peroxidation\,inhibition\,activity }}\left( \% \right) = [(A_{\text{C}} - A_{\text{S}} )/A_{\text{C}} ] \times 100\%$$

where *A*_S_ is the absorbance value with samples and *A*_C_ is the absorbance value without samples.

### Supercoiled pBR322 plasmid DNA assay

According to the references of Lin et al., and Zuo et al. [27, 28], supercoiled pBR322 plasmid DNA assay was measured. Briefly, 10 mM AAPH in PBS (pH 7.4) was added 100 ng of pBR322 DNA to a final volume of 25 μL in microcentrifuge tubes at 37 °C for 1 h. The 25 μL solution contains 15 μL AAPH, 5 μL DNA, 5 μL antioxidants. Five microliter distilled water was used in the absence of antioxidants. After incubation, 2 μL 10× loading buffer were mixed with the samples, loaded into a 0.8% agarose gel. The agarose gel was electrophoresed in 1× TAE gel buffer for 75 min (20 mA, 50 V). Using the Bio-Rad Gel Doc XR system (New York, America), the gels were then photographed under UV transillumination. DNA strand breaks were evaluated. The amount of supercoiled DNA was quantified by the Bio-Rad Quantity One software.

One-way ANOVA was used to analyze the differences among means, and statistically significant was considered by a P < 0.05 value (SPSS version 13.0, SPSS).

## Results

### Tyrosinase activity assay

**Fig. 1:**
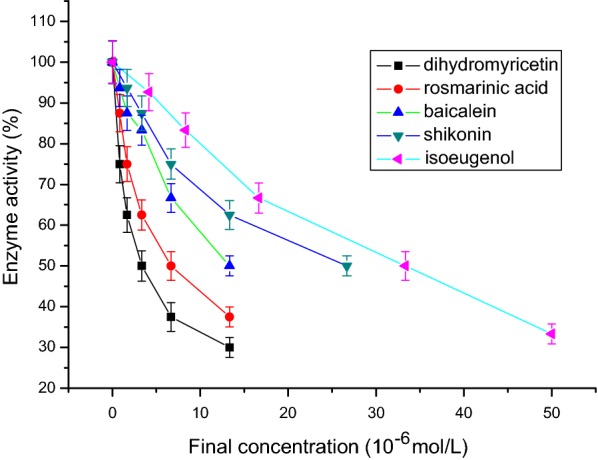
The inhibition effects of isoeugenol, shikonin, baicalein, rosmarinic acid, and dihydromyricetin on the diphenolase activity of mushroom tyrosinase. The IC_50_ values of the five compounds on the tyrosinase diphenolase activity were respectively 33.33 μmol/L, 26.67 μmol/L, 13.33 μmol/L, 6.67 μmol/L, and 3.33 μmol/L (n = 5, P < 0.05)

The substrate of tyrosinase for the diphenolase activity assay was l-DOPA. The results showed that a group of lines with different slopes passing through the origin was the progress curve of enzyme reaction. The slope indicated the diphenolase activity. In the progress of oxidation of l-DOPA, the lag period did not exist. Isoeugenol, shikonin, baicalein, rosmarinic acid, and dihydromyricetin exhibited, with dose dependence, inhibitory effect on tyrosinase diphenolase activity. The IC_50_ values of the five compounds on the tyrosinase diphenolase activity were respectively 33.33 μmol/L, 26.67 μmol/L, 13.33 μmol/L, 6.67 μmol/L, and 3.33 μmol/L (n = 5, P < 0.05, Fig. 1; Table 1). The order of activity was: isoeugenol < shikonin < baicalein < rosmarinic acid < dihydromyricetin. Therefore, the five compounds had obvious inhibitory effects on the tyrosinase diphenolase activity. The order of activity was very consistent with the docking score between tyrosinase and compounds.

**Table 1:** The IC_50_ values of flavonoids

	Isoeugenol	Shikonin	Baicalein	Rosmarinic acid	Dihydromyricetin
Tyrosinase diphenolase activity (μmol/L)	33.33	26.67	13.33	6.67	3.33
DPPH free radical (μmol/L)	101.6	83.2	58.6	28.5	12.4
ABTS free radical (μmol/L)	36.36	27.27	9.09	6.82	3.41
Hydroxyl free radical (μmol/L)	32.5	18.3	11.6	8.3	4.2
Superoxide free radical (μmol/L)	38.2	31.5	16.1	12.3	7.6
Lipid peroxidation (μmol/L)	25.1	16.67	12.5	8.33	6.25

### Inhibition mechanism on the diphenolase activity of tyrosinase

**Fig. 2:**
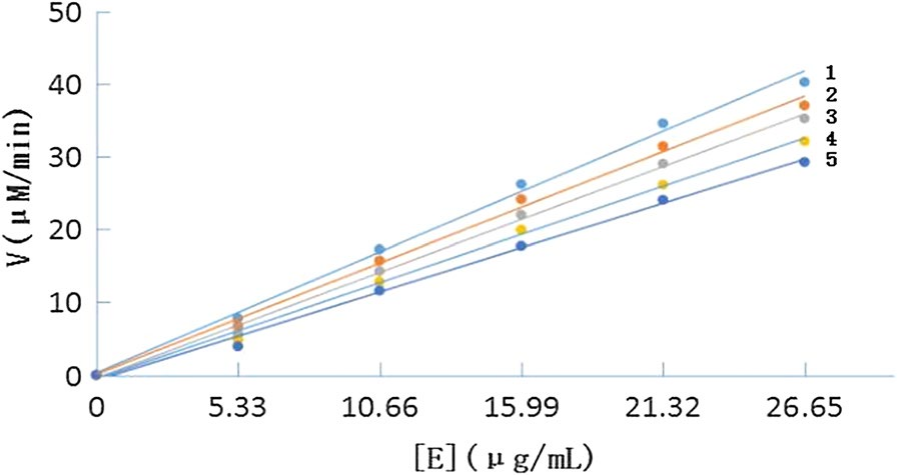
Determination of the inhibitory mechanism of shikonin on mushroom tyrosinase. The results showed that shikonin was reversible inhibitor of tyrosinase for the oxidation of l-DOPA. l-DOPA = l-3,4-dihydroxyphenylalanine

The inhibitory mechanism of isoeugenol, shikonin, baicalein, rosmarinic acid, and dihydromyricetin on tyrosinase for oxidation of l-DOPA was examined. The relationship between the concentration of five compounds and enzyme activity was examined. The inhibitory mechanism of shikonin on tyrosinase was tested. As shown in Fig. 2, at different inhibitor concentrations, the plots of enzyme activity versus the enzyme concentration gave a family of straight lines, which all passed through the origin. The final concentration of shikonin for curves 1–5 was respectively 0 μmol/L, 3.3 μmol/L, 6.67 μmol/L, 13.33 μmol/L, and 26.67 μmol/L. The presence of an inhibitor resulted in the inhibition of enzyme activity, but did not reduce the amount of enzyme. The inhibitors showed the same behavior. The results exhibited that isoeugenol, shikonin, baicalein, rosmarinic acid, and dihydromyricetin were reversible inhibitors of tyrosinase diphenolase.

**Fig. 3:**
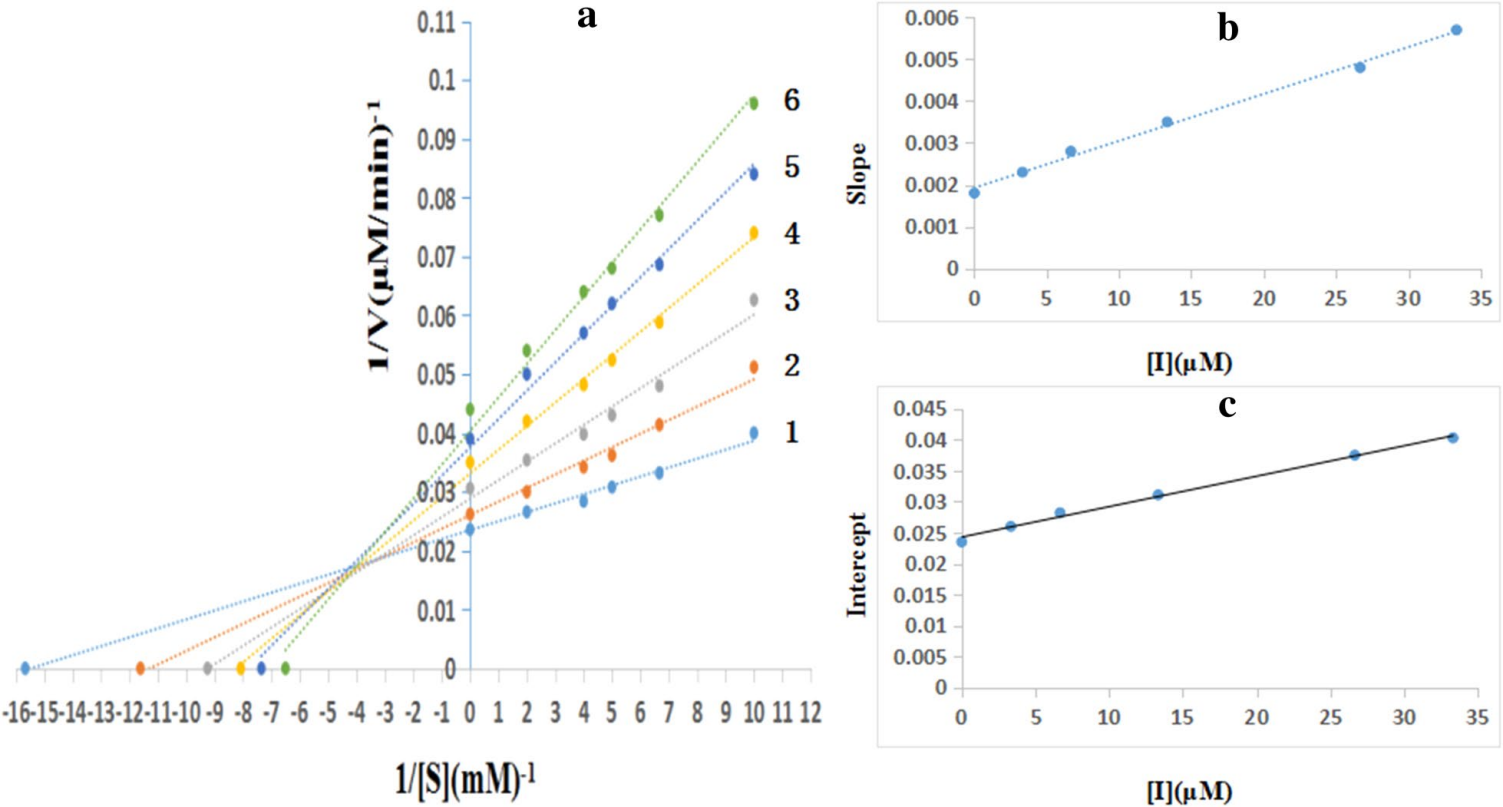
**a** Lineweaver–Burk plots for the inhibition of shikonin on mushroom tyrosinase for the oxidation of l-DOPA. **b** The plot of slope versus the concentration of shikonin for determining the inhibition constants K_I_. K_I_ = 19 μmol/L. **c** The plot of intercept versus the concentration of shikonin for determining the inhibition constants K_IS_. K_IS_ = 48.6 μmol/L.K_I_ = equilibrium constant for inhibitor binding with free enzyme; K_IS_ = enzyme–substrate complex; l-DOPA = l-3,4-dihydroxyphenylalanine

By Lineweaver–Burk double-reciprocal plots for the inhibition tyrosinase diphenolase, the inhibition type of the five compounds was examined. The enzyme kinetics in the presence of shikonin are shown in Fig. 3. The final concentration of shikonin for curves 1–6 was respectively 0 μmol/L, 3.3 μmol/L, 6.67 μmol/L, 13.33 μmol/L, 26.67 μmol/Land 33.33 μmol/L. Lineweaver–Burk double-reciprocal plots were the plots of 1/v versus 1/[S]. A family of straight lines intercepted in the second quadrant, which indicated that shikonin was a competitive–uncompetitive mixed type inhibitor (Fig. 3a). It indicated that shikonin can combine with not only enzyme–substrate complexes, but also free enzymes. From a plot of the slope (K_m_/V_mapp_) versus the concentration of the inhibitor, K_I_ was measured (Fig. 3b). From a plot of the vertical intercept (1/V_mapp_) versus the concentration of inhibitor, K_IS_ was measured (Fig. 3c). The values of K_I_ and K_IS_ were determined as 19.0 μM and 48.6 μM, respectively. By contrast, isoeugenol was the same inhibitor type as shikonin, and the inhibitor constants (K_I_ and K_IS_) were determined as 25.6 μM and 64.7 μM, respectively. Baicalein was the same inhibitor type as shikonin, and the inhibitor constants (K_I_ and K_IS_) were determined as 16.5 μM and 38.4 μM, respectively. Rosmarinic acid was the same inhibitor type as shikonin, and the inhibitor constants (K_I_ and K_IS_) were determined as 14.3 μM and 29.8 μM, respectively. Dihydromyricetin was the same inhibitor type as shikonin, and the inhibitor constants (K_I_ and K_IS_) were determined as 10.26 μM and 23.6 μM, respectively.

### Molecular docking

**Fig. 4:**
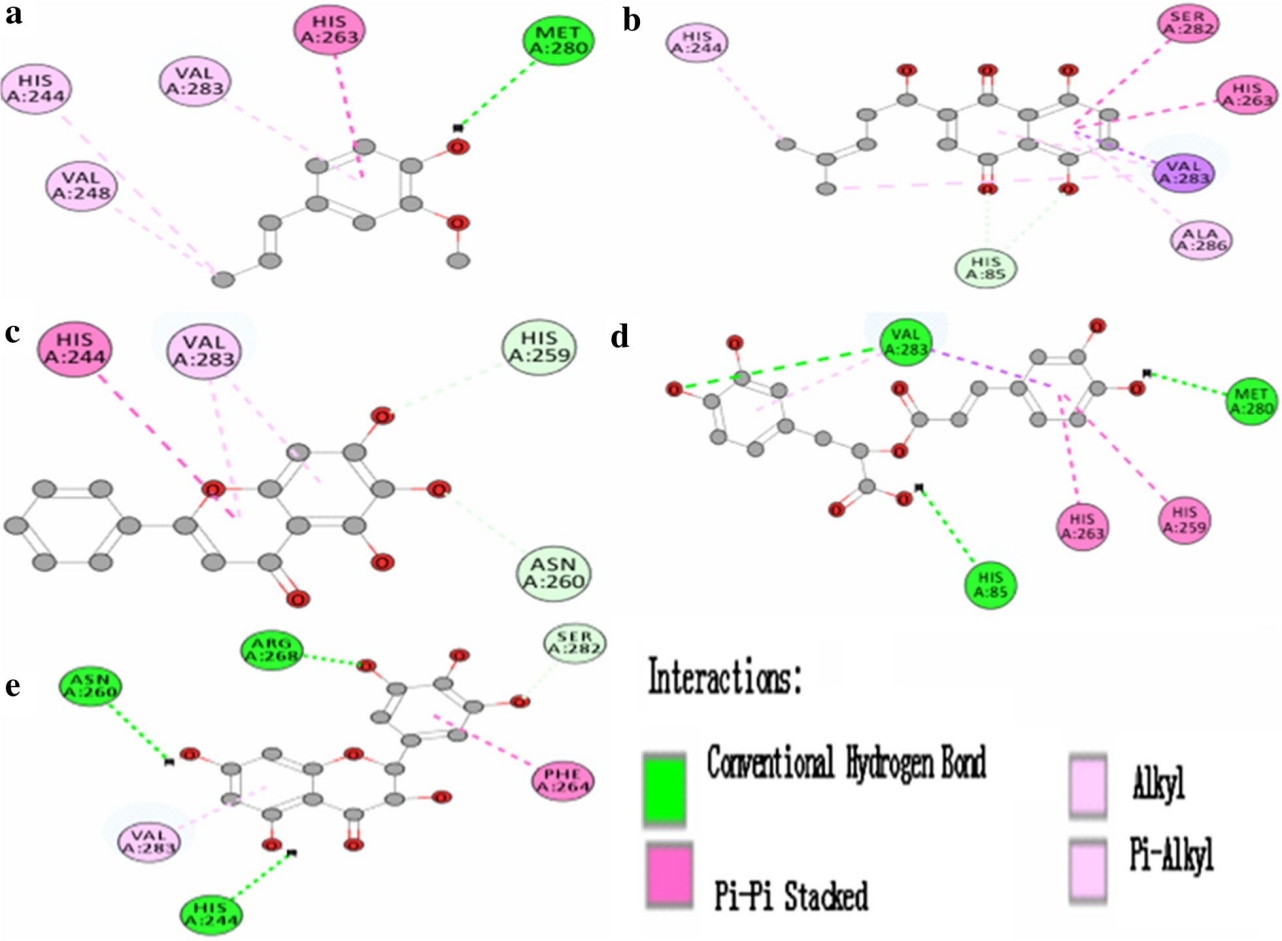
Docking simulations 2D diagram of binding position and binding mode between tyrosinase and compound isoeugenol (**a**), shikonin (**b**), baicalein (**c**), rosmarinic acid (**d**), and dihydromyricetin (**e**), respectively

Figure 4 shows that docking simulations colored 2D-representations of binding mode and binding position between tyrosinase and compound isoeugenol (a), shikonin (b), baicalein (c), rosmarinic acid (d), and dihydromyricetin (e), respectively. The binding interactions between tyrosinase and compound include mainly the pi–pi stacked, conventional hydrogen bond, pi–alkyl, and alkyl. Molecular docking results implied that the compounds could interact with the amino acid residues in the active center of tyrosinase.

The docking score between tyrosinase and compound isoeugenol, shikonin, baicalein, rosmarinic acid, and dihydromyricetin was 33.14, 36.13, 37.93, 44.56, 50.98, respectively. The order of activity was: isoeugenol < shikonin < baicalein < rosmarinic acid < dihydromyricetin. The order of activity was very consistent with the experimental results (Fig. 1). Docking score indicates the interaction affinity between enzyme and ligand by the optimized algorithm, which helps to speculate the scope of inhibitory activity. The main significance of docking score is the evaluation index for quick preliminary screening compounds. In this paper, based on the docking score, the inhibit tyrosinase activity of five typical compounds was verified by the experiments in vitro.

**Fig. 5:**
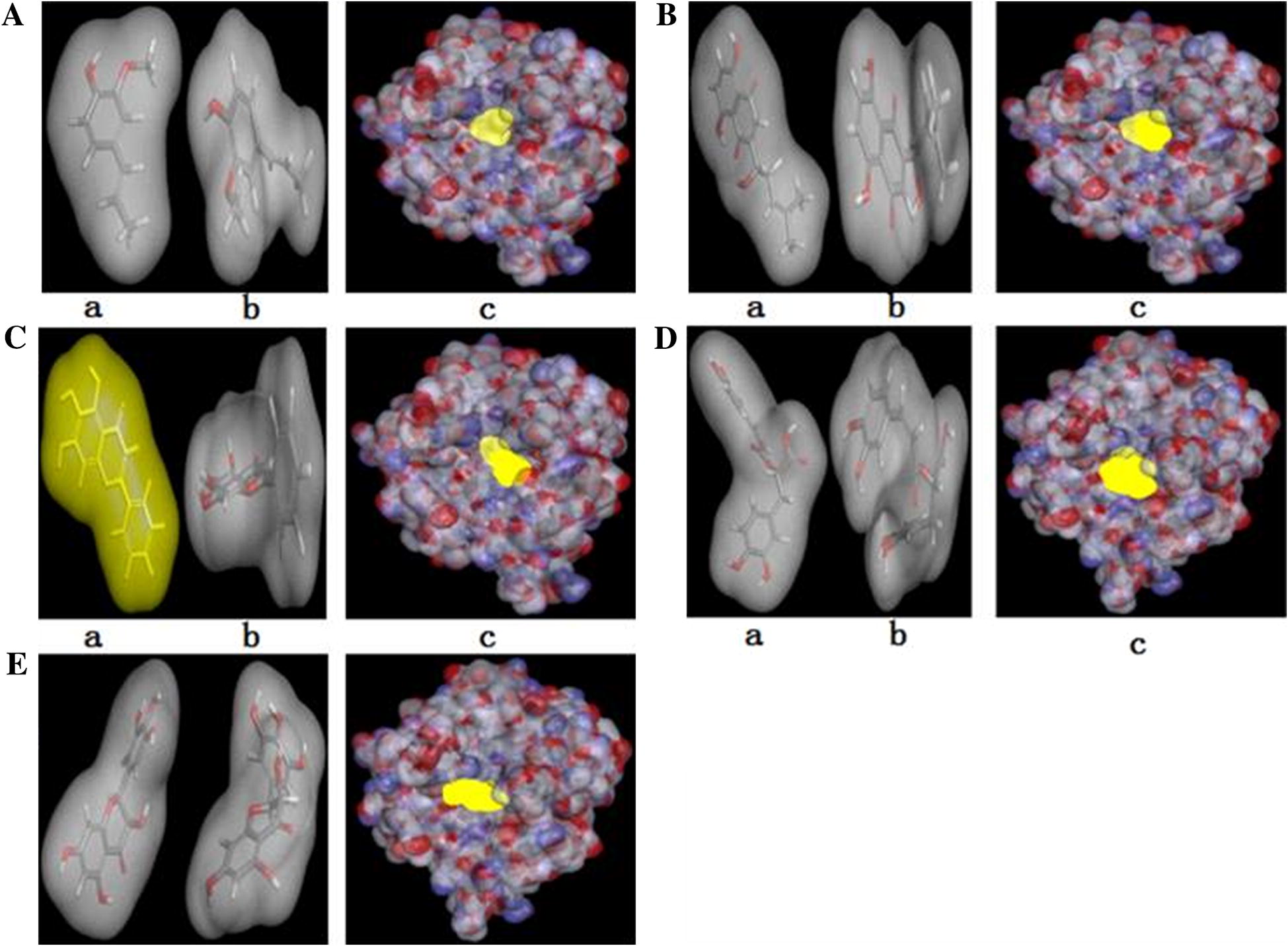
Colored 3D-representations of the protein–ligand complex showed that surface and conformation changes of compounds before (a) and after (b) docking into tyrosinase, and docking simulation of compound isoeugenol (**A**), shikonin (**B**), baicalein (**C**), rosmarinic acid (**D**), and dihydromyricetin (**E**), respectively, in the hydrophobic pocket of tyrosinase (c)

Figure 5 shows that docking simulations of conformational changes and binding position between tyrosinase and inhibitors. Colored 3D-representations of the protein–ligand complex showed that surface and conformation changes of compounds before (a) and after (b) docking into tyrosinase. Docking simulations of binding position of compound isoeugenol (A), shikonin (B), baicalein (C), rosmarinic acid (D), and dihydromyricetin (E), respectively, in the hydrophobic pocket of tyrosinase (c), which indicates the inhibition mechanism on the diphenolase activity of tyrosinase.

The combination mode and binding sites of tyrosinase and five typical compounds were studied by molecular simulation. The results showed that these compounds enter the hydrophobic activity cavity of tyrosinase, change the enzyme conformation, which in turn affect the catalytic activity. The hydrogen bonds between Met 280, Val 283, His 85 residues and compounds, the pi–pi bonds between Phe 264, His 244, His 259, or His 263 and compounds or pi–alkyl bonds between Val 283, Val 248 and compounds, may be related to the identification and fix the ligand and tyrosinase. Besides phenolic hydroxyls, the scaffold components of different compounds may also effect on their antityrosinase activities. Particularly, different hydrophobic groups may have significant contribution to binding with the hydrophobic cavity of the target proteins. The molecular docking results showed the detailed information and the visual evidence of the binding position between the tyrosinase and inhibitors. The similar binding position and binding mode may be the similar inhibition mechanism. However, without any experimental evidence, the developed models will be too early to be applicable for antityrosinase activity of compounds. The result of Seo [29] indicated that CDOCKER and CDOCKER interaction energies of quercetin and its analogues were decreased by C151W mutation whereas benzoic acid and its analogues did not lower the energies. In particular, the results illustrated the blockage of pi–pi stacked or pi–alkyl interactions between quercetin and quercetin-4′-methyl ether and His154 or Val132. These results indicate that the influence of Cys 151 residue of Keap1 keeps on the interaction between compounds and Keap1 protein.

### DPPH free radical scavenging activity

**Fig. 6:**
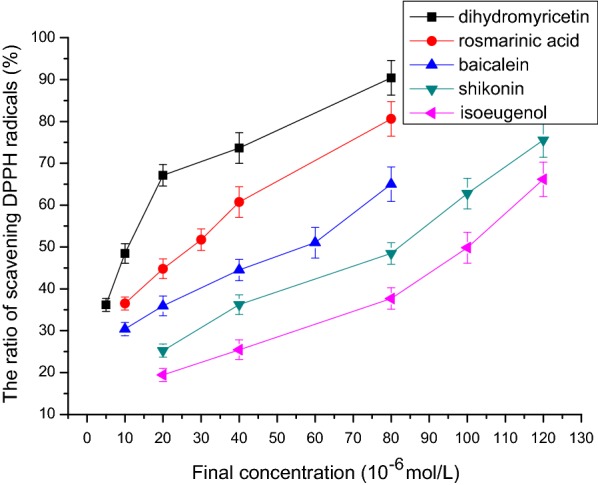
The relationship between final concentration and the ratio of scavenging DPPH radicals. The IC_50_ values of DPPH free radical scavenging capacity of isoeugenol, shikonin, baicalein, rosmarinic acid, and dihydromyricetin were respectively 101.6 μmol/L, 83.2 μmol/L, 58.6 μmol/L, 28.5 μmol/L, and 12.4 μmol/L (n = 3, P < 0.05). *DPPH* 1,1-diphenyl-2-picrylhydrazyl

Figure 6 shows that isoeugenol, shikonin, baicalein, rosmarinic acid, and dihydromyricetin had obvious DPPH free radical scavenging activity. The IC_50_ values of DPPH free radical scavenging capacity of isoeugenol, shikonin, baicalein, rosmarinic acid, and dihydromyricetin were respectively 101.6 μmol/L, 83.2 μmol/L, 58.6 μmol/L, 28.5 μmol/L, and 12.4 μmol/L (n = 3, P < 0.05, Table 1). The order of activity was: isoeugenol < shikonin < baicalein < rosmarinic acid < dihydromyricetin.

The result of Zhu [17] indicated that IC_50_ of DPPH radical scavenging activity of rosmarinic acid extract was 5.5 ± 0.2 μg/mL, and IC_50_ of α-glucosidase inhibitory activity was 0.23 ± 0.01 mg/mL. The result of Liu [30] showed that IC_50_ of DPPH radical scavenging activity of the dihydromyricetin–lecithin complex was 22.60 μg/mL. The result of Xu [31] showed that the scavenging capacity of hydroxyl radical (·OH), superoxide radical (O_2_·), and alkane radical (ROO·) for dihydromyricetin was 83.9%, 90.0%, and 63.9% respectively.

### ABTS free radical scavenging activity

**Fig. 7:**
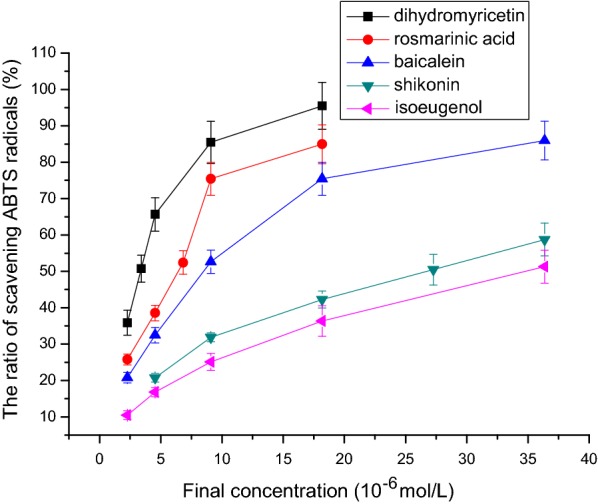
The relationship between final concentration and the ratio of scavenging ABTS radicals. The IC_50_ values of ABTS free radical scavenging capacity of isoeugenol, shikonin, baicalein, rosmarinic acid, and dihydromyricetin were respectively 36.36 μmol/L, 27.27 μmol/L, 9.09 μmol/L, 6.82 μmol/L, and 3.41 μmol/L (n = 3, P < 0.05). ABTS = 2,2′-azino-bis-(3-ethylbenzothiazoline-6-sulphonic acid)

Figure 7 shows that isoeugenol, shikonin, baicalein, rosmarinic acid, and dihydromyricetin had obvious ABTS free radical scavenging activity. The IC_50_ values of ABTS free radical scavenging capacity of isoeugenol, shikonin, baicalein, rosmarinic acid, and dihydromyricetin were respectively 36.36 μmol/L, 27.27 μmol/L, 9.09 μmol/L, 6.82 μmol/L, and 3.41 μmol/L (n = 3, P < 0.05, Table 1). The order of activity was: isoeugenol < shikonin < baicalein < rosmarinic acid < dihydromyricetin.

### Hydroxyl free radical scavenging activity

**Fig. 8:**
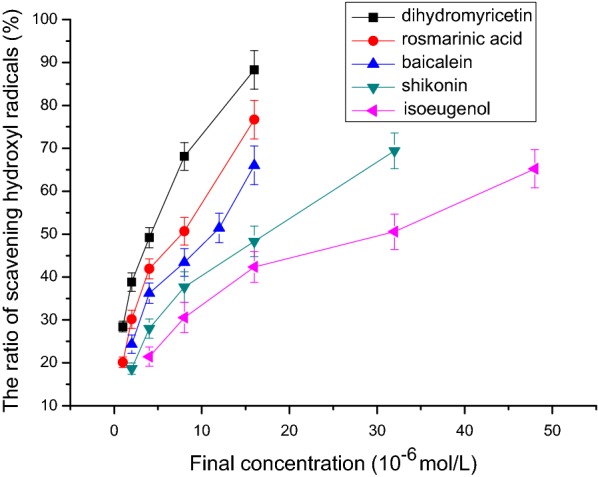
The relationship between final concentration and the ratio of scavenging hydroxyl radicals. The IC_50_ values of hydroxyl free radical scavenging capacity of isoeugenol, shikonin, baicalein, rosmarinic acid, and dihydromyricetin were respectively 32.5 μmol/L, 18.3 μmol/L, 11.6 μmol/L, 8.3 μmol/L, and 4.2 μmol/L (n = 3, P < 0.05)

Figure 8 shows that isoeugenol, shikonin, baicalein, rosmarinic acid, and dihydromyricetin had obvious hydroxyl free radical scavenging activity. The IC_50_ values of hydroxyl free radical scavenging capacity of isoeugenol, shikonin, baicalein, rosmarinic acid, and dihydromyricetin were respectively 32.5 μmol/L, 18.3 μmol/L, 11.6 μmol/L, 8.3 μmol/L, and 4.2 μmol/L (n = 3, P < 0.05, Table 1). The order of activity was: isoeugenol < shikonin < baicalein < rosmarinic acid < dihydromyricetin.

### Superoxide free radical scavenging activity

**Fig. 9:**
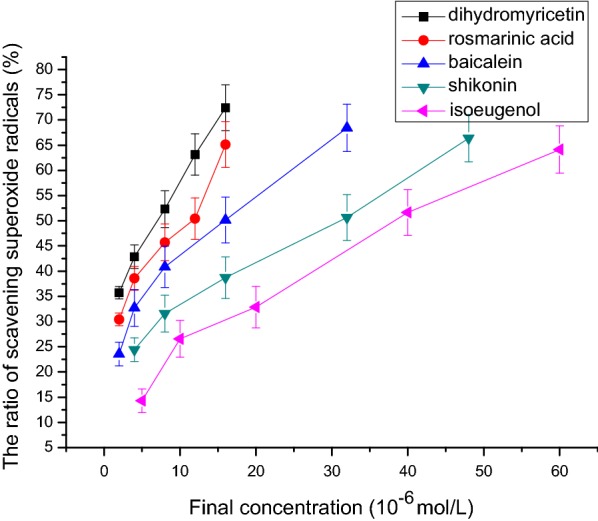
The relationship between final concentration and the ratio of scavenging superoxide radicals. The IC_50_ values of superoxide free radical scavenging capacity of isoeugenol, shikonin, baicalein, rosmarinic acid, and dihydromyricetin were respectively 38.2 μmol/L, 31.5 μmol/L, 16.1 μmol/L, 12.3 μmol/L, and 7.6 μmol/L (n = 3, P < 0.05)

Figure 9 shows that isoeugenol, shikonin, baicalein, rosmarinic acid, and dihydromyricetin had obvious superoxide free radical scavenging activity. The IC_50_ values of superoxide free radical scavenging capacity of isoeugenol, shikonin, baicalein, rosmarinic acid, and dihydromyricetin were respectively 38.2 μmol/L, 31.5 μmol/L, 16.1 μmol/L, 12.3 μmol/L, and 7.6 μmol/L (n = 3, P < 0.05, Table 1). The order of activity was: isoeugenol < shikonin < baicalein < rosmarinic acid < dihydromyricetin.

### Lipid peroxidation assay in liver mitochondria in vitro

**Fig. 10:**
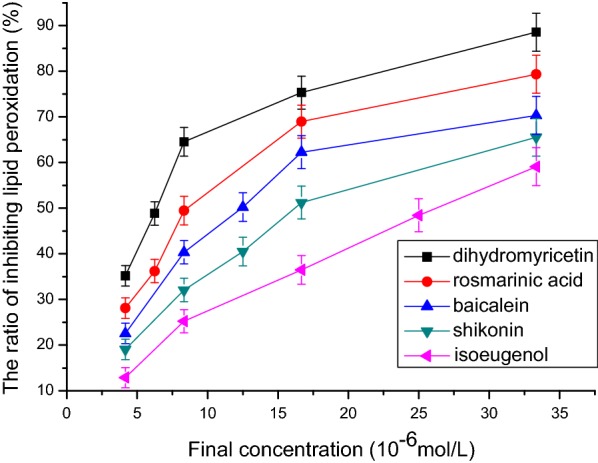
The relationship between final concentration and the ratio of inhibiting lipid peroxidation. The IC_50_ values of inhibiting lipid peroxidation of isoeugenol, shikonin, baicalein, rosmarinic acid, and dihydromyricetin were respectively 25.1 μmol/L, 16.67 μmol/L, 12.5 μmol/L, 8.33 μmol/L, and 6.25 μmol/L (n = 3, P < 0.05)

Figure 10 shows that isoeugenol, shikonin, baicalein, rosmarinic acid, and dihydromyricetin had obvious activity of inhibiting lipid peroxidation. The IC_50_ values of inhibiting lipid peroxidation of isoeugenol, shikonin, baicalein, rosmarinic acid, and dihydromyricetin were respectively 25.1 μmol/L, 16.67 μmol/L, 12.5 μmol/L, 8.33 μmol/L, and 6.25 μmol/L (n = 3, P < 0.05, Table 1). The order of activity was: isoeugenol < shikonin < baicalein < rosmarinic acid < dihydromyricetin.

### Supercoiled pBR322 plasmid DNA assay

**Fig. 11:**
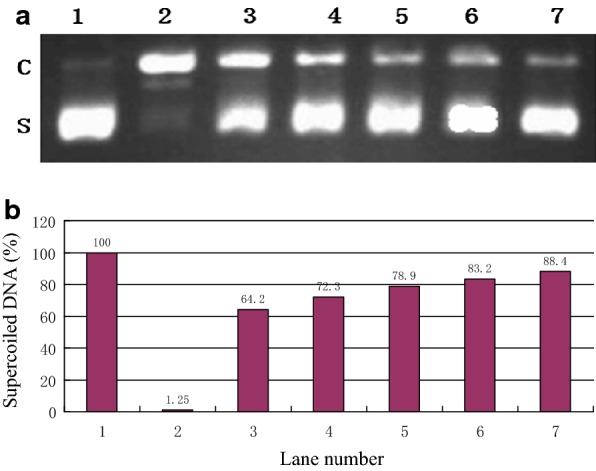
**a** Agarose gel electrophoretic patterns of supercoiled pBR322 plasmid DNA converted into the open circular by AAPH in the presence or absence of samples (10 μM). **b** The effects of samples on supercoiled pBR322 plasmid DNAconverted into the open circular by AAPH in the presence or absence of samples(10 μM). Lane 1: control (native pBR322 DNA, without AAPH); Lane 2: AAPH; Lane 3: AAPH + isoeugenol; Lane 4: AAPH + shikonin; Lane 5: AAPH + baicalein; Lane 6: AAPH + rosmarinic acid; Lane 7: AAPH + dihydromyricetin. The density of the supercoiled DNA form was quantified by Quantity One (Bio-Rad). Data are the average of three determinations; *C* open circular, *S* supercoil, *AAPH* 2,2′-azobis(2-methylpropionamidine)dihydrochloride

Figure 11a shows that in the absence of AAPH, the plasmid DNA was mainly supercoiled. The supercoiled form of plasmid DNA was changed into the linear forms and open circular with the addition of 10 mM AAPH. In the presence of 10 μM compounds, the amount of supercoiled form increased, but the amount of the linear and circular forms decreased. The amount of supercoiled plasmid DNA was quantified by the Bio-Rad Quantity One software. Figure 11b shows the observed values. Thus, these compounds exhibited protection against free radical injury induced by AAPH in a dose-dependent manner. The order of inhibition activity was: isoeugenol < shikonin < baicalein < rosmarinic acid < dihydromyricetin.

An index of DNA damage is used as the change of bacteriophage or plasmid DNA from the supercoiled form to the linear forms. Strand breaks in pBR322 DNA can be caused by the presence of AAPH [32].

## Discussion

Isoeugenol is the major constituent of *E. caryophyllata* Thunb. The result of Hubungan [33] indicated that antioxidant activities in the following orders: butylated hydroxytoluene (BHT) > mannich product of isoeugenol > isoeugenol > mannich product of eugenol > eugenol. The result of Ko [34] indicated that demethyldi-isoeugenol inhibited Fe^2+^-induced lipid peroxidation. It also scavenged superoxide anion generated by peroxyl radical (ROO.) derived from AAPH.

Shikonin is the major constituent of *Arnebia euchroma*(*Royle*)*Johnst*. The observed results revealed that shikonin demonstrated higher reducing ability (0.431%), and deoxy-shikonin showed maximum inhibition (0.440%) to DPPH-radical scavenging assay.

Baicalein is the major constituent of *Rheum officinale*. The results of Nishioka [35] revealed that baicalein can inhibit the express of human intestinal sucrase in the Caco-2 cells. The results of Tsai [36] revealed that baicalein can protect against the acute lung injury induced by lipopolysaccharide in rats. The results of Jeli [37] revealed that baicalein exhibit good inhibitory activities of both production of cytokine IL-6 and tyrosine kinase.

Rosmarinic acid can inhibit the enzymatic browning of fruits and vegetables. The result of Ha [38] showed that rosmarinic acid possess mushroom tyrosinase inhibitory activities (IC_50_ of 4.0 μM). The result of Ding [39] showed that rosmarinic acid methyl ester can inhibit tyrosinase, and reduce the melanin contents in B16 cells. The result of Fujimoto [40] showed that rosmarinic acid afforded a highly tyrosinase-inhibitory active product. Rosmarinic acid has antioxidant and prooxidant activities. The result of Sánchez-Campillo [41] indicated that rosmarinic acid can be used as a good photo-protective agent.

Zhao et al. [42] evaluated the antioxidant properties of Citri Exocarpium Rubrum based on its DPPH free radical scavenging activity, ferric ion reducing antioxidant power (FRAP) and trolox equivalent antioxidant capacity (TEAC) assays. Bivariate correlation analysis revealed correlations between the characteristic peaks and the antioxidant activities of the samples. *Sambucus williamsii* Hance (*Jiegumu*) is traditionally used in Chinese medicine to treat bone and joint diseases. The major phytochemicals are phenolic acids, lignans, and terpenoids. This compounds may have the antioxidant, anti-inflammatory, bone fracture healing, and anti-osteoporotic effects [43].

Tyrosinase (EC 1.14.18.1) play a key role in melanin biosynthesis [44]. Due to the over expression of tyrosinase, excessive melanin leads to melasma and age spots [45]. Tyrosinase is responsible for the browning of vegetables and fruits in the food industry, which results in reduced market value and shorter product shelf life [46]. Increased attention has also drawn to the applications of antioxidants and tyrosinase inhibitors as preservatives in skin-protective ingredients in cosmetics and in the food industry. On the other hand, ROS could induce oxidative damage of proteins and DNA, and peroxidation of membrane lipids. Lipid peroxidation will generate malondialdehyde (MDA), and do harm to cells [47]. It may be useful in diets to obtain properly antioxidants.

## Conclusion

In conclusion, isoeugenol, shikonin, baicalein, rosmarinic acid, and dihydromyricetin exhibited good antityrosinase activities. These compounds also exhibited good antioxidant effects on lipid peroxidation, supercoiled pBR322 plasmid DNA, and DPPH, ABTS, hydroxyl, or superoxide free radical scavenging activity. The different molecular structures lead to the different antityrosinase and antioxidant activities. The activity order is isoeugenol < shikonin < baicalein < rosmarinic acid < dihydromyricetin. The results showed the compounds with more phenolic hydroxyls have more antioxidant and antityrosinase activities. This was the first study of molecular docking for modeling the antityrosinase activity of compounds. This was also the first study of the lipid peroxidation inhibition activity of compounds in liver mitochondria induced by Fe^2+^/vitamin C(Vc) system in vitro, the protective effects on supercoiled pBR322 plasmid DNA. In a word, the results support the use of compounds as the new anti-aging candidate drugs, cosmetic materials and food additives.

## Additional file

**Additional file 1.** Minimum Standards of Reporting Checklist.

## Abbreviations

ROS: reactive oxygen species
l-DOPA: l-3,4-dihydroxyphenylalanine
DPPH: diphenyl-2-picrylhydrazyl
TBA: thiobarbituric acid
ABTS: 2, 2′-azino-bis (3-ethylbenzothiazoline-6-sulfonic acid)
AAPH: 2,2′-azobis(2-methylpropionamidine)dihydrochloride
DMSO: dimethyl sulfoxide
IC50: inhibitory concentration 50
